# Oxidative stress, inflammation, and steatosis elucidate the complex dynamics of HgCl_2_ induced liver damage in *Channa punctata*

**DOI:** 10.1038/s41598-024-59917-4

**Published:** 2024-04-22

**Authors:** Shefalee Singh, Shikha Dwivedi, Adeel Ahmad Khan, Anamika Jain, Shraddha Dwivedi, Kamlesh Kumar Yadav, Indrani Dubey, Abha Trivedi, Sunil P. Trivedi, Manoj Kumar

**Affiliations:** 1 Environmental Toxicology and Bioremediation Laboratory (ETBL), Department of Zoology, University of Lucknow, Lucknow, 226007 India; 2 Department of Zoology, DBS College, Kanpur, Uttar Pradesh 208006 India; 3 Department of Zoology, Government Degree College, Bakkha Kheda, Unnao, 209801 India; 4 Department of Zoology, Government Degree College, Haripur-Nihastha, Raebareli, 229208 India; 5 Department of Zoology, Mahatma Jyotiba Phule Rohilkhand University, Bareilly, Uttar Pradesh 243006 India

**Keywords:** Zoology, Environmental sciences

## Abstract

Water bodies are highly pollution-prone areas in which mercury (Hg) is considered as a major menace to aquatic organisms. However, the information about the toxicity of mercuric chloride (HgCl_2_) in a vital organ such as the liver of fish is still inadequate. This study aimed to assess the impact of mercuric chloride (HgCl_2_) exposure on the liver of *Channa punctata* fish over 15, 30, and 45 days, at two different concentrations (0.039 mg/L and 0.078 mg/L). Mercury is known to be a significant threat to aquatic life, and yet, information regarding its effects on fish liver remains limited. The results of this study demonstrate that exposure to HgCl_2_ significantly increases oxidative stress markers, such as lipid peroxidation (LPO) and protein carbonyls (PC), as well as the levels of serum glutamic-oxaloacetic transaminase (SGOT) and serum glutamic pyruvic transaminase (SGPT) in the fish. Additionally, the transcriptional and protein analysis of specific genes and molecules associated with necroptosis and inflammation, such as ABCG2, TNF α, Caspase 3, RIPK 3, IL-1β, Caspase-1, IL-18, and RIPK1, confirm the occurrence of necroptosis and inflammation in the liver. Histopathological and ultrastructural examinations of the liver tissue further reveal a significant presence of liver steatosis. Interestingly, the upregulation of PPARα suggests that the fish's body is actively responding to counteract the effects of liver steatosis. This study provides a comprehensive analysis of oxidative stress, biochemical changes, gene expression, protein profiles, and histological findings in the liver tissue of fish exposed to mercury pollution in freshwater environments.

## Introduction

Heavy metal contamination is a widespread problem, with a particular emphasis on mercury, in aquatic ecosystems in India. Mercury pollution is a critical concern due to its adverse impacts on the environment and human health. India is a significant contributor to global mercury emissions, and various sources, such as industrial waste, compact fluorescent lamps (CFLs), and groundwater, have resulted in elevated mercury levels in both organisms and humans. The urgency of comprehending and addressing the repercussions of mercury pollution on aquatic ecosystems and public health is needed. Many studies highlight that mercury affects various organs and systems in aquatic organisms, emphasizing the need for environmental and health measures to mitigate contamination. The gravity of mercury pollution in India and its significant implications, emphasizing the importance of monitoring and regulating mercury emissions to protect both the environment and human well-being is largely obscure hitherto. In the list of the world's biggest sources of environmental mercury (Hg), India stands at number two with an approximate discharge of 144.7 tonnes of Hg/year^1^. Hg contamination in India is pointing towards lethal conditions due to the release of unwanted pollutants having a range of Hg from 0.058 to 0.268 mg/L. This is higher than the permissible Indian and WHO limits of 0.001 mg/L for drinking water and 0.01 mg/L for industrial effluents. Large numbers of mercury poisoning cases have been reported in the recent past, which are largely ignored. The leading anthropogenic and natural release of Hg exists as Mercuric (II) chloride (EPA, 2020).

Mercury enters in the aquatic regimes through various natural (geothermal springs and volcanic eruptions) and anthropogenic activities (coal-fired power plants and waste incineration).Once in the water, mercury undergoes various transformations. Inorganic mercury, such as HgCl_2_, can be converted into methyl mercury (MeHg), a highly toxic form that tends to bioaccumulate in aquatic organisms. Fish can absorb mercury through their gills, skin, and diet. Inorganic mercury is transformed into organic forms like methylmercury by bacteria in water, and this form is readily taken up by aquatic organisms. As fish consume contaminated prey or come into contact with contaminated water, mercury accumulates in their tissues, particularly in the liver and muscles.Mercuric chloride harms fishes by inducing oxidative stress, damaging cellular structures, particularly in the liver, and triggering inflammatory responses, ultimately compromising fish health and survival^2^. Additionally, mercury accumulates in fish tissues, disrupting physiological processes and posing risks to both aquatic ecosystems and human consumers. Since Hg is absorbed and bioaccumulated in fish, it harms aquatic regimes as well as human health through biomagnification. The elevated levels of Hg can lead to mortality of aquatic fauna, ultimately decline the number of species, majorly fish communities, and badly impact overall aquatic biodiversity^3^. Thus, the information on Hg toxicity in aquaculture has become very important in research. A large number of investigations have been performed on the effect of heavy metals on aquatic fauna^4,5^ but, despite being the imperative organ of xenobiotic metabolism in fish, there is not sufficient information available on the potential risk ofHgCl_2_pollution induced liver steatosis in fish.

Several studies have documented that Hg imbalances ROS production and its removal by the antioxidant system, which is known as an oxidative stress response^6^. Oxidative stress plays a significant role in initiating heavy metal toxicity in fish^7–9^. Assessing the activity of lipid peroxidation, protein carbonyl, and biomarker enzymes in liver tissue reflects the expanse of oxidative stress^10^. There are an ample number of studies describing oxidative stress induced by heavy metals as responsible for necroptosis, inflammation, and apoptosis in different organisms^11^. Oxidative stress-mediated necroptosis and inflammation inducing liver steatosis in fish remain largely obscure hitherto under long-term exposure to HgCl_2_. To fill this research gap, the present study was conducted, which illustrates the above-mentioned parameters coupled with the transcriptional and translational profile of certain necroptosis and inflammation related genes in freshwater food fish *Channa punctata*. This is an empirical study to explore the effects of HgCl_2_ exposure on ultrastructural perturbations, enzymatic, biochemical, and molecular levels in the liver of fish, which will greatly extend our understanding of the pathways involved in systemic toxicity of HgCl_2_ in fish. The study is necessary due to the environmental urgency, public health concerns, data gaps, application of advanced research methods, the need for effective policy measures, and the importance of conserving aquatic biodiversity. It serves as a vital step in addressing the multifaceted challenges posed by mercury contamination in India and beyond. This study is innovative in deriving howHgCl_2_impacts *Channa punctata*, delving into oxidative stress, necroptosis, and inflammation pathways in the fish liver. By addressing gaps in understanding HgCl_2_-induced toxicity, it provides unique insights into molecular responses, gene expressions, and protein profiles, fostering a more comprehensive understanding of mercury's multifaceted effects on fish physiology. This enriches scientific literature in the field of aquatic toxicology.

## Materials and methods

### Test chemical

S.D Fine-chem. Ltd., Mumbai, India manufactured HgCl_2_ was procured from a scientific chemical dealer.

### Chemicals used

Methane sulfonate, ethylenediaminetetraacetic acid, potassium per magnet, dimethyl sulfoxide, hydrochloric acid, 2,4-dinitrophenylhydrazine, trichloroacetic acid, ethanol, ethyl acetate, sodium hydroxide, guanidine hydrochloride, trizol, chloroform, isopropanol, DEPC water, thiobarbituric acid, acrylamide, bisacrylamide, tris base, sodium dodecyl sulphate, ammonium persulphate, Temed, sodium chloride, Tween20, bovine serum albumin, bromophenol blue, glycerol, mercapto ethanol, methanol, sodium carbonate, copper sulphate, potassium sodium tartarate, Folins reagent, acetone, glutaraldehyde, osmium tetraoxide, DPX, Bouins fluid, alcohol.

### Determination of median lethal concentration of HgCl_2_

In order to determine the 96-h LC_50_ of HgCl_2_ for *Channa punctata*, standard protocols outlined in the American Public Health Association (APHA) 2017^12^ and the Organization for Economic Co-operation and Development (OECD) guidelines for fish acute bioassays (OECD203, 92/69/EC, method C1) were followed. The study involved five aquaria, each containing four acclimatized fish with an average weight of 30 ± 3.0 g and length of 14.5 ± 1.0 cm. These fish were exposed to five concentrations of HgCl_2_ on a logarithmic scale (0.1, 1.0, 10, 100, and 1000 mg/L) for 96 h in a semi-static bioassay system, while maintaining a fish loading rate of 4 g/L of water^13^. The observed lethality range was noted to be between 0.1 and 1.0 mg/L of HgCl_2_. Subsequently, a logarithmic series of ten nominal concentrations (0, 0.2, 0.4, 0.6, 0.8, 1.0, 1.2, 1.4 mg/L) was employed to determine the LC_50_ value. The LC_50_ values for HgCl_2_ at 96 h were calculated using the probit analysis method^14^.

### Sample size calculation

The sample size was calculated using the software GPower 3.1^15–19^, based on ANOVA for repeated measures, effects between subjects, a significance level of 5%, a power of 90%, and an expected medium effect size (0.5), yielding 135 fishes.

### Experimental design

*Channa punctata* (30 ± 5.0 g; 15 ± 1.0 cm) were collected from water bodies of Lucknow and its suburbs (26° 55′ N, 80° 59′ E), Uttar Pradesh, India. Specimens were brought to the laboratory and prophylactic treatment (0.05% KMNO_4_) was given for 3–5 min, to remove exterior contagions if any^20^. Acclimatization of 135 fish for 15 days was done in 160 L well aerated glass aquaria (100 × 40 × 40 cm^3^) filled with 100 L of 15 days aged tap water^12^. Fish were fed two times a day with commercial aquarium food pellets (Perfect Companion Group Company Limited, Thailand) at the rate of 3% of the body weight per day^21^. A day before the start of the experiment feeding was stopped^22^. The experimental design consisted of three groups, each replicated in triplicate, with 15 fish in each group. Group I served as the control, while Group II was exposed to a dose of 0.039 mg/L of HgCl_2_ (equivalent to 96 h-LC_50_/20). Group III received a dose of 0.078 mg/L of HgCl_2_ (equivalent to 96 h-LC_50_/10). These specific concentrations were selected to represent controlled conditions, a sub-lethal dose, and a higher but still sub-lethal dose, respectively, allowing for the assessment of potential effects on the fish over designated exposure period. No mortality was reported during the experiment but some behavioural changes were observed like hyper activeness, surfacing, gulping, and jumping in the fish. The aquaria were cleaned and water was renewed^23^ daily to prevent waste and debris^24^. On each sampling day (15, 30, and 45d), three fish from every replicate were anesthetized by using tricaine methane sulfonate (MS-222; 0.3 g/L Sigma Aldrich E10521)^25^, a heparinized syringe was used to withdraw blood from the caudal blood vessel and stored in vials coated with EDTA (1.8 mg/mL) for ROS examination. Fish were dissected longitudinally from the ventral side and its liver was excised for evaluation of oxidative stress, biochemical, molecular changes, and tissue histology. Samplings were done as per OECD 2019, guidelines. The study was carried out following the ARRIVE guidelines.

### Evaluation of cell viability

Cell viability was assessed using the trypan blue exclusion assay at three time points: 15, 30, and 45 days. In this assay, 50 μL of the cell suspension was mixed with 0.4% trypan blue solution in 1:1 (v/v) ratio, and viable cells were quantified using a hemocytometer^26,27^. Samples with a cell viability exceeding 85% underwent additional analysis using the single-cell gel electrophoresis (SCGE) assay and ROS production. Viable cells, which expelled the dye and exhibited a round, shiny appearance, remained unstained, whereas non-viable cells retained the blue stain (Supplementary Fig. S3).

Percentage of viable cells were counted as follows

$${\text{Viable \,cells }}\% \, = {\text{total \,number \,of\, viable\, cells \,per\, mL \,of \,aliquot}}/{\text{total\, number of\, cells\, per \,mL \,of \,aliquot }} \times\, { 1}00$$

### ROS production

A fluorescent dye 2′, 7′-dichlorodihydrofluorescein (20 µM, DCFH-DA; Sigma Aldrich, USA) was used to quantify ROS level in the blood of test fish^2,28^. The fluorescence intensity of stained blood cells depicts the amount of ROS generated. Image J software (version 1.50, USA) was used to measure the corrected total cell fluorescence.

### DNA fragmentation assay

DNA fragmentation was measured by SCGE/comet assay^27^. The assay was conducted using a three-layer technique with minor adjustments. In summary, approximately 15 μL of cell suspension containing about 20,000 cells was mixed with 85 μL of 0.5% low melting-point agarose (LMPA) and layered onto a frosted glass slide pre-coated with 200 μL of 1% normal agarose. After solidification, a third layer of 100 μLLMPA was added. Slides were immersed in a lysing solution overnight at 4 °C. Subsequently, they were subjected to alkaline electrophoresis in fresh cold alkaline electrophoresis buffer for 20 min at 4 °C. Electrophoresis was performed at 15 V (0.8 V/cm) and 300 mA. Slides were neutralized with Tris buffer, stained with ethidium bromide, and comet images were analyzed using an image analyzer system to quantify DNA damage based on percent tail DNA (% tail DNA).

### The activity of the liver biomarker enzymes

At 3000 rpm blood was centrifuged for 10 min to separate serum. In the serum, the activity level of SGOT and SGPT was analyzed by kinetic method using the commercial kit (Robonik India Pvt. Ltd, Navi Mumbai). Absorbance was measured using a Shimadzu UV-1700 pharma spec UV–VIS spectrophotometer. Due to the oxidation of NADH to NAD, there was a significant decrease in absorbance measured at 340 nm, which is directly proportional to the activity of SGOT and SGPT in the sample. Its activity is expressed in Units/L**.**

### Estimation of LPO

The LPO in liver tissue was estimated by following the methodology of Buege and Aust, (1978)^29^. The breakdown of polyunsaturated fatty acids results in the formation of malondialdehyde, which serves as a convenient index for determining the extent of the peroxidation reaction.

### Estimation of protein carbonyls in the liver

The method of Levine et al. (1990)^30^ was used to estimate the activity level of protein carbonyls. A protein-DNP hydrazone moiety is produced due to the reaction of dinitrophenylhydrazine (DNPH) with protein carbonyl groups, which was detected through a spectrophotometer.

### Transcriptional analysis of targeted genes

TRIzol method^31^ was used to isolate total RNA from liver tissue.NanoDrop 2000/2000c (Thermo Scientific, USA) was used to ascertain the quality and quantity of the isolated RNA. cDNA synthesis was performed using Revert Aid H Minus Synthesis Kit (#K1632; Thermo Scientific, USA) as per manufacturer's lab manual, and alteration in the mRNA levels of ABCG2, TNF α, Caspase 3, RIPK 3, IL-1β, PPAR-α, Caspase-1, IL-18 and RIPK1 (Table 1) in response to HgCl_2_ treatment was quantified by a CFX96™ Real-Time PCR System (qRT-PCR) (C1000 Touch™ Thermal Cycler, Bio-Rad, USA) using SYBR Green Master mix (#A25742; Thermo Scientific, USA). The qRT-PCR data were analyzed using the relative gene expression 2^−ΔΔCt^method^32^ and the results were depicted as the fold change in mRNA transcripts standardized to the endogenous reference gene β-actin. Bands of amplified DNA were also pictured by 1% agarose gel electrophoresis (Bio-Rad, USA).

**Table 1:** Primer sequences used in the study.

Name of gene	Primer sequence	Accession number	Primer efficiency (%)	Conditions	Product length	R2 value	Pearson’s coefficient
ABCG2	(F) CTTGAAGGAACGTTGATGTG (R)CCCAGATGGAAGAAAGGAAA	NM_001042775.1	98.2	(F) Tm-51.9 °C, GC content-40.0% (R) Tm-51.4 °C, GC content-45.0%	180	0.999	0.999
TNF α	(F) GCTTCCTCAGACCACGGAAA (R) CAGCGATTGTCCTGAAGGGT	NM_001024447.1	94.5	(F) Tm-57.2 °C, GC content-55.0% (R) Tm-57.4 °C, GC Content- 55.0%	93	0.998	0.998
Caspase 3	(F) GCCAGACAAAGCGATGCAAA (R) AGCCCAGCTGTGAGAAAGTC	NM_131877.3	94.1	(F) Tm-56.8 °C, GC content-50.0% (R) Tm-57.3 °C, GC Content- 55.0%	472	0.991	0.995
RIPK 3	(F) TAGCGTCTGGCATTGGGTTT (R) GACATTGCATCACAGTCGGC	XM_001343791.5	95.6	(F) Tm-57.1 °C, GC Content-50.0% (R) Tm-56.8 °C, GC Content- 55.0%	761	0.994	0.996
IL-1β	(F) GAATGAAGCACATCAAACCC (R) GCAGCTCGAAGTTAATGATG	NM_212844.2	97.2	(F) Tm-52.1 °C, GC content-45.0% (R) Tm-51.5 °C, GC Content- 45.0%	106	0.999	0.999
PPAR-α	(F) AACAAATCCAAAGCACGAAC (R) GAACGTTAACAATGCTCTCC	NM_001102567.1	98.1	(F) Tm-51.9 °C, GC Content- 40.0% (R) Tm-51.4 °C, GC Content- 45.0%	296	0.993	0.996
Caspase-1	(F) CGGCATGTGCAGAATGGAAC (R) AGAGTCCGGGGAACAGGTAG	MG957992.1	96.3	(F) Tm-57.1 °C, GC Content- 55.0% (R) Tm-58.2 °C, GC Content- 60.0%	121	0.997	0.998
IL-18	(F) AGGCGTCTCATTATTGTGTT (R) CCTCCTGGTAGTTGATAACG	AY389462.1	95.2	(F) Tm-51.9 °C, GC Content- 40.0% (R) Tm-52.2 °C, GC Content- 50.0%	140	1	1
RIPK1	(F) TGGACCAAACCATCAGCTCC (R) TGCCACATGATTTGCTCCCT	NM_001043350.1	91.4	(F) Tm-57.4 °C, GC Content- 55.0% (R) Tm-57.3 °C, GC Content- 50.0%	157	0.993	0.996
β-actin	(F) GTGCCCATCTAGAGGGTTA (R)AAGGAAGGAAGGCTGGAAGA	AF057040.1	98.5	(F) Tm-56.3 °C, GC Content- 55.0% (R) Tm-55.8 °C, GC Content- 40.0%	73	0.992	0.995

### Protein expression

Protein expression was assessed by western blotting method for TNF α, Caspase 3, RIPK1, and β-Actin in liver tissue of control and 15, 30, and 45 days of HgCl_2_ treated (Group II and III) *C. punctata*. TNF α, Caspase 3, RIPK1, and β-Actin proteins were identified and expressions were evaluated, total protein extricated from hepatic tissue of fish was lysed in RIPA buffer having 1X protease inhibitor, followed by estimation of protein concentrations by the method of Lowry et al., (1951)^33^. About 20–50 μg of protein was loaded in each lane and allowed for separation by 15% SDS-PAGE and from gel it was transferred to a polyvinylidene difluoride (PVDF) membrane (Bio-Rad Cat. #162-0177). After being blocked with 3% BSA in 1× Tris-buffered saline Tween (TBST) buffer, the membrane was blotted overnight with the primary antibodies at 4 °C and then incubated with the horseradish peroxidase-conjugated secondary antibody (1:5000, Elabscience E-AB-1003) for 1 h at 37 °C. The proteins were distinguished using the Clarity™ Western ECL Substrate (Bio-Rad Cat. # 170-5061), and digital photographs were obtained using a gel-imaging system (Molecular Imager^R^ChemiDoc™ XRS + with Image Lab™ Software Bio-Rad). The antibodies used for the experiments were: anti-RIPK1 (1:2000, E-AB-18284), anti- TNF α (1:2000, E-AB-33121), anti- Caspase-3 (1:2000, E-AB-66940) and anti- β actin (1: 4000, E-AB-30422).

### Histopathology of the liver

For histological observation, Graff et al., 2022^34^ were followed, the liver tissue was cleaned with normal saline solution and kept for 48 h in Bouin’s fluid for fixation then washed with 70% ethyl alcohol repeatedly for 4–5 days to get rid of extra Bouin’s fluid. Tissue was gradually dehydrated in 50%, 70%, 90%, and 100% ethanol, then embedded in paraffin wax. For tissue sectioning Yorco Precision Rotary Microtome, India (YSI062) was used. Finally, sections were stained with hematoxylin and counterstained with eosin for 2 min (Ratn et al., 2018^35^) and examined under an oil immersion microscope (Nikon Corporation K 12432) with 10/40× magnification of the objective lens. Images were analyzed using ImageJ software; version ImageJ bundled with 64-bit Java 1.8.0_172.

### Ultra-structural analysis

For scanning electron microscopic (SEM) study tissue was fixed in 2.5% glutaraldehyde solution for a day at 4 °C, and post fixing tissue was kept in 1% osmium tetroxide solution for two hours at 4 °C. Dehydration was performed through a graded series of acetone (50%, 70%, 90%, and 100%) after fixation. Tissue was dried with a critical point drier and then fixed on a metal stub and sputter-with gold plating (about 20 nm thick) and observed under the SEM (JSM 6490). Histopathological observations were analyzed by following Farrel et al., 2008^36^.

### Statistical analysis

The triplicate of each group was carried out and data were depicted as mean ± standard error mean (S.E.M.). SPSS software (version 20.0, SPSS Company, Chicago, USA) was used to evaluate the data. The significance (p < 0.05) of data was evaluated using one-way ANOVA with Tukey's post hoc test. Regression and correlation analysis were also performed among various physiological parameters.

### Ethical statement

Experimentation was permitted by the Committee for Control and Supervision of Experiments on Animals (CCSEA), Ministry of Environment and Forests, Government of India under registration no.1861/GO/Re/S/16/CCSEA, and approved by the institutional ethical committee of University of Lucknow, Lucknow, India. All methods were performed according to the relevant guidelines and regulations.

## Results and discussion

The 96 h-LC_50_ value of HgCl_2_ for *Channa punctata* was 0.783 mg/L with a 95% confidence limit of 0.724 mg/L (Lower) and 0.853 mg/L (Upper). Fish were exposed to two sub-lethal concentrations for chronic study (96 h-LC_50_/20) and (96 h-LC_50_/10) of HgCl_2_. Physicochemical parameters of water such as the potential of hydrogen (pH), the concentration of dissolved oxygen, hardness, temperature, and alkalinity were within the prescribed limits for the endurance of the fish (APHA, 2017)^11^, and no major variations among these parameters were observed (Table 2).

**Table 2:** Physicochemical parameters table on mercuric chloride toxicity.

Physicochemical parameters	Groups	Exposure period		
		15 days	30 days	45 days
Temperature	Group I	24.9 ± 0.35	23.83 ± 0.31	24.46 ± 0.68
	Group II	23.23 ± 1.18	22.5 ± 0.35	23.13 ± 0.51
	Group III	23.23 ± 1.03	23.83 ± 0.85	23.46 ± 0.91
pH	Group I	7.20 ± 0.05	7.16 ± 0.14	7.23 ± 0.12
	Group II	7.30 ± 0.05	7.2 ± 0.05	7.16 ± 0.03
	Group III	7.23 ± 0.08	7.1 ± 0.25	7.16 ± 0.03
Alkalinity	Group I	74.66 ± 1.05	73.75 ± 0.83	77.17 ± 0.69
	Group II	74.13 ± 1.10	75.13 ± 0.58	77.68 ± 0.66
	Group III	75.7 ± 1.12	76.78 ± 1.10	74.9 ± 1.08
Hardness	Group I	73.32 ± 0.17	74.08 ± 0.09	74.51 ± 0.21
	Group II	73.79 ± 0.22	75.14 ± 0.02	76.32 ± 0.31
	Group III	73.44 ± 0.32	74.78 ± 0.06	76.23 ± 0.07
Dissolved oxygen	Group I	7.07 ± 0.15	7.03 ± 0.01	6.67 ± 0.18
	Group II	6.49 ± 0.28	7.28 ± 0.05	6.85 ± 0.02
	Group III	7.49 ± 0.34	6.46 ± 0.19	6.50 ± 0.19

**Figure 1:**
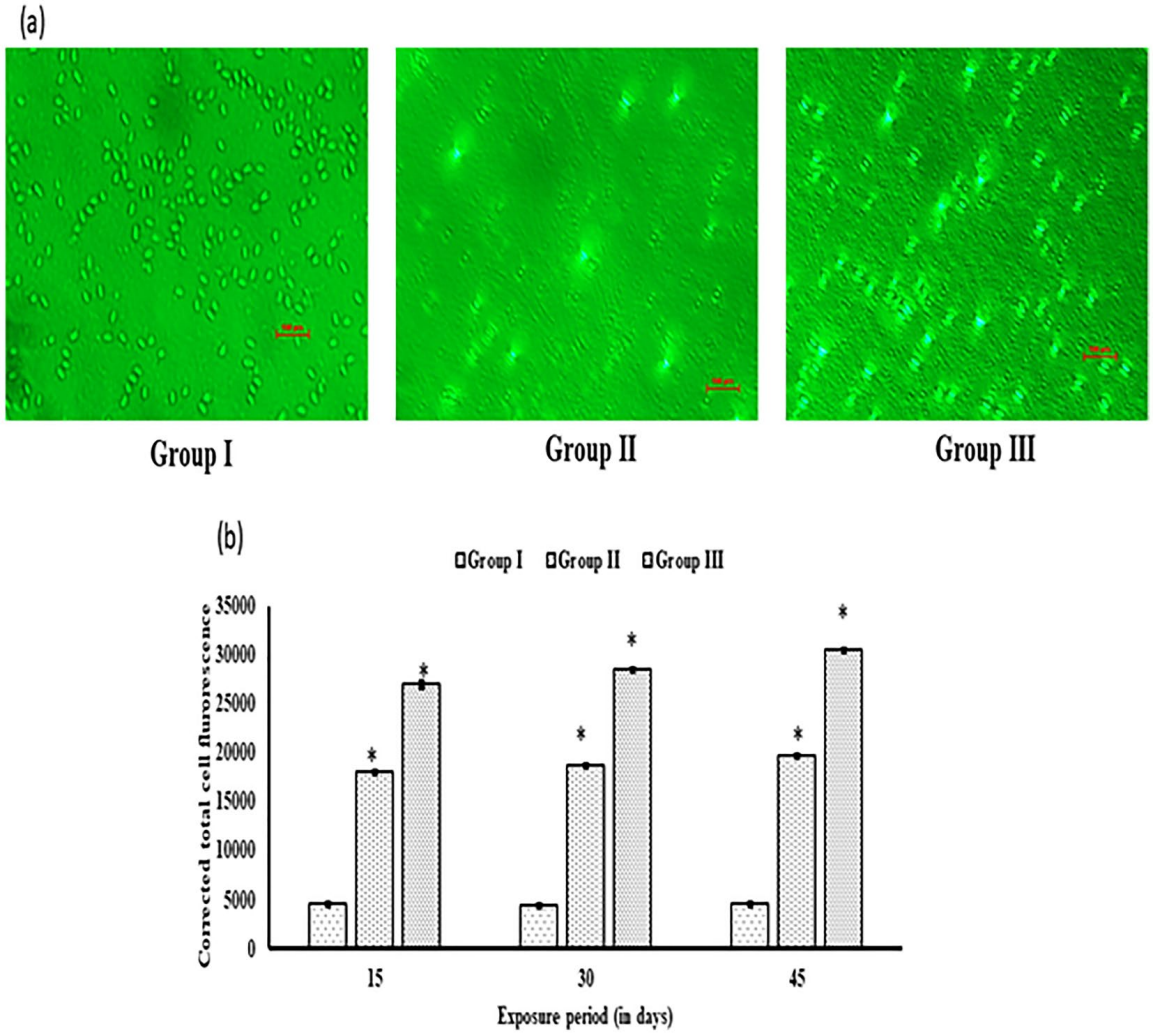
(**a**) ROS production after 45 d of exposure in fish *C. punctata* induced by HgCl_2._ (**b**) The significant (p < 0.05) increment in ROS in HgCl_2_treated groups II and III as compared to group I. The data are expressed as mean ± S.E.M. (n = 3 fishes) of three replicates of each group. (mean ± SD, n = 3 fishes of three replicates of each group) (as represents the significant (p < 0.05) difference from the control).

**Figure 2:**
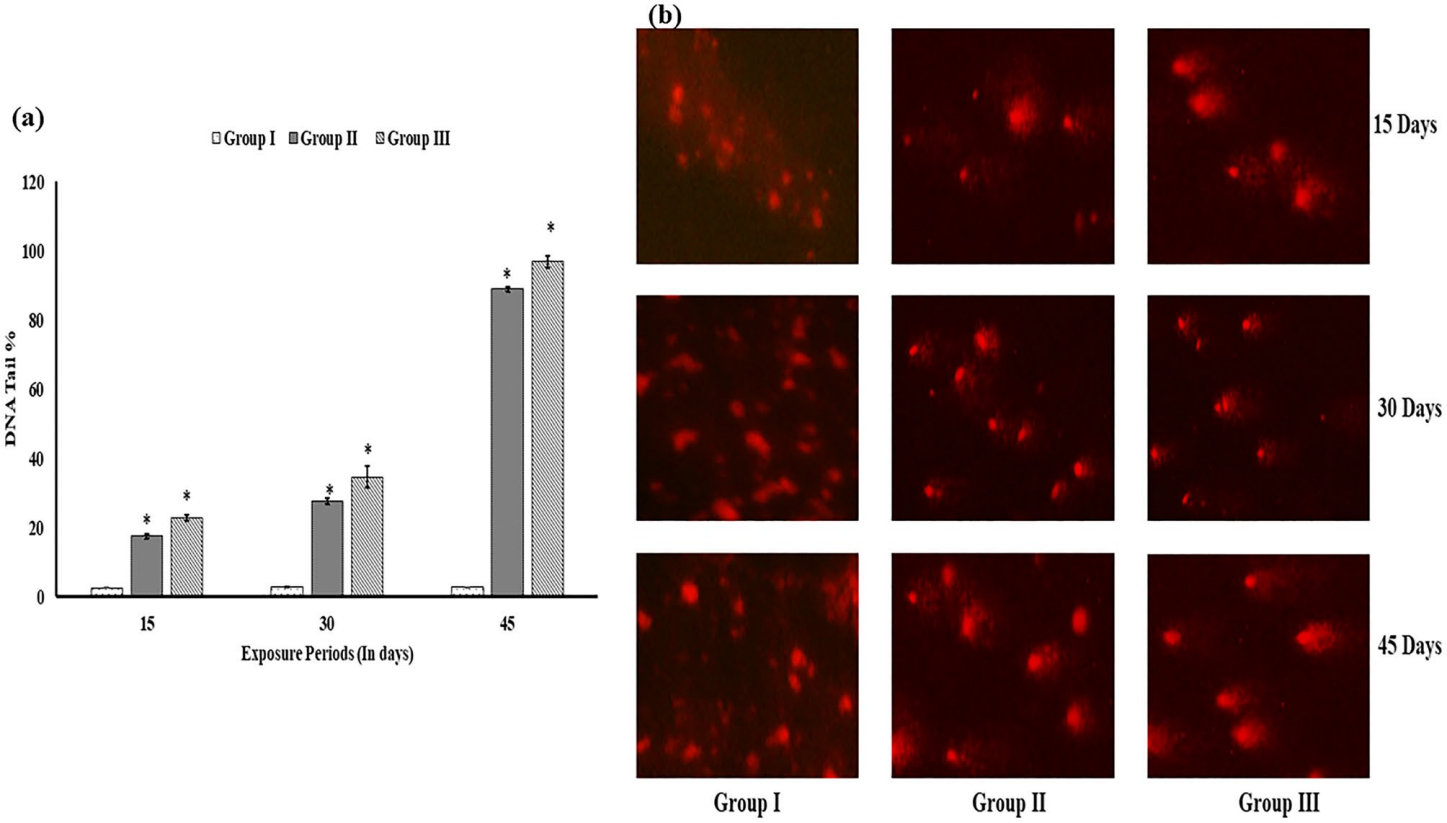
(**a**) The percentage of DNA tail length in blood cells of *Channa punctata* was measured for groups I, II, and III after 15,30 and 45 days of exposure. (**b**) Comet assay results of blood samples from *Channa punctata* illustrating the levels of DNA damage in erythrocytes following 15, 30 and 45 days of exposure to HgCl_2_.

ROS production was significantly (p < 0.05) augmented following an increase in the concentration of HgCl_2_ with respect to control (Fig. 1b). An increment in ROS level was recorded in both the HgCl_2_intoxicated groups in a time-dependent manner. The ROS generation was maximum in group III after 45 d of the exposure period (Fig. 1a).Furthermore, the percentage of tail DNA, indicating DNA damage, was assessed in blood cells from both control and exposed groups (Fig. 2a). Figure 2b illustrates the DNA strand breaks resulting from exposure to HgCl_2_ in *C. punctata* fish. Group III exhibited the highest DNA damage on day 45 compared to the control group. Fish exposed to HgCl_2_ in groups II and III showed significantly elevated DNA damage levels compared to the control group.

**Figure 3:**
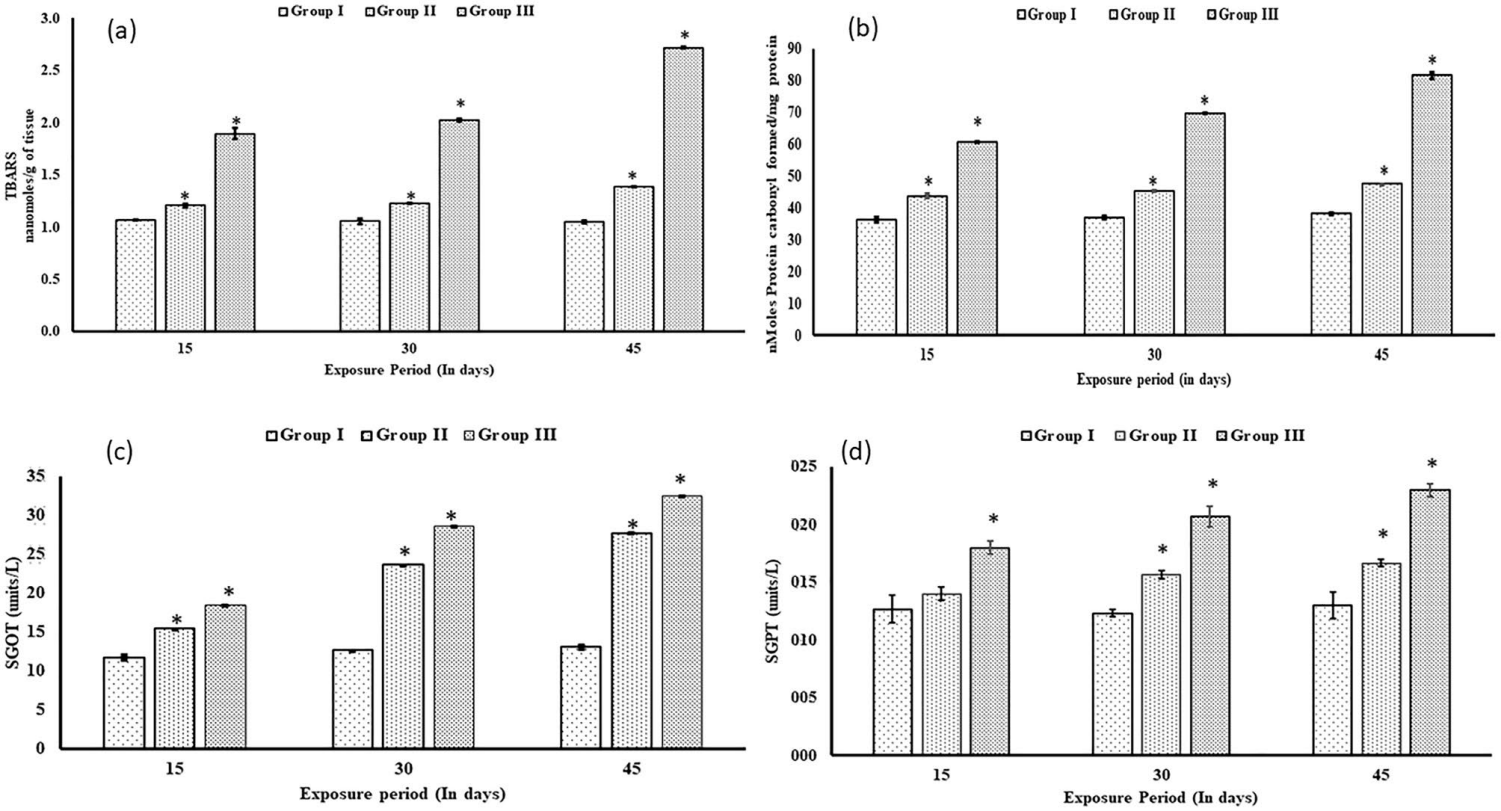
Effect of two sublethal concentrations of Mercuric chloride on (**a**) lipid peroxidation (**b**) protein carbonyl (**c**) SGOT and (**d**) SGPT as compared to the control of test fish *C. punctata*. (mean ± SD, n = 3 fishes of three replicates of each group) (asterisk represents the significant (p < 0.05) difference from the control).

In general, the activity of lipid peroxidation (TBARS), protein carbonyls, and liver marker enzymes (SGOT and SGPT) were increased significantly (p < 0.05) in all groups treated with sublethal concentrations of HgCl_2_ (Fig. 3a–d). Lipid peroxidation was the highest (2.72 ± 0.009) in exposed group III (maximum concentration of HgCl_2_) as compared to the control (1.90 ± 0.05) after 45 days of the exposure period. In group III the level of protein carbonyls was 81.0 ± 0.87 nmol carbonyls/mg protein which was significantly higher than the control group, where it was 60.8 ± 0.14 nmol carbonyls/mg protein. All the treated groups (groups II and III) show significant (p < 0.05) differences in the level of protein carbonyl as compared to the control group in a time-dependent manner. The activity level of SGOT and SGPT was significantly (p < 0.05) altered after every exposure period in treated groups as compared to the control group. The highest increment in SGOT (32.5 ± 0.00) and SGPT (0.023 ± 0.00) was found in group III as compared to the control 18.5 ± 0.001 and 0.018 ± 0.001 respectively after 45 days.

The metabolism of heavy metals often leads to the formation of reactive intermediates like ROS, which possess high toxicity and can directly cause DNA breakage or hinder the repair of oxidized DNA bases^37^. While organisms possess antioxidant defence systems to safeguard tissues against oxidative damage, excessive ROS production beyond the capacity of these defence mechanisms can result in cellular and DNA breakage. Oxidative damage to DNA, triggered by ROS production during processes like respiration, has been identified as a significant factor contributing to the extensive and varying levels of DNA damage observed in the gill cells of *C. punctata*^5^. Exposure to mercuric chloride in aquatic environments makes fish species like trout, salmon, and tilapia especially vulnerable^38^. In this study mercuric chloride can induce oxidative stress mediated DNA strand breaks which causes genotoxicity in fish *Channa punctata*. Similarly, Trivedi et al., 2022^2^ have reported that mercuric chloride can cause genotoxicity in targeted fish. The resulting oxidative damage disrupts cellular functions, initiating lipid peroxidation in biomolecules like lipids and proteins. LPO, along with protein carbonyls serves as a marker for oxidative stress, indicating potential cell death pathways. LPO is a method in which electrons are removed from lipids by free radicals and later produce reactive species. The LPO acts as a cell death signal to induce different cell death pathways and is also capable of damaging phospholipids directly^39^. Cell damage can be induced by the oxidation of lipids and proteins. So, the LPO and PCs have been identified as markers for lipid and protein oxidation in various organs of the body^40^. PC groups have been used as biomarkers of oxidative stress^41^. Increased LPO and PCs in fish liver and kidney can lead to impaired organ function, inflammation, and potential ecological and health risks^42^. This study is in harmony with Sharma, (2017)^43^ who has documented similar results and evaluated the lead toxicity in the muscle of fish *C. punctata*. Furthermore, increased ROS and oxidative stress in fish liver initiates detrimental effects on cellular functions. This disturbance between ROS and antioxidant defences is reflected by elevated activities of enzymes like SGOT and SGPT in this study. The increased SGOT and SGPT levels serve as indicators of disrupted cellular processes, potentially reflecting instabilities in amino acid synthesis and transamination. These enzymatic changes underscore the physiological impact of oxidative stress on fish health, emphasizing the interconnected relationship between ROS-induced damage and the enzymatic responses observed in SGOT and SGPT activities. In this study, increased activity of SGOT and SGPT was observed at sub-lethal exposure of HgCl_2_.The elevated level of either SGOT or SGPT suggests an increased synthesis of amino acids or an increased transamination process from fatty acids or glucose during HgCl_2_ intoxication. In*C. punctata* sub-lethal concentrations of chromium and copper increased the level of SGOT and SGPT activity ^37,38^. In fish, increased SGOT and SGPT activities can be indicative of liver and kidney diseases. These conditions can affect the overall health and survival of the fish, potentially leading to impaired growth, reproduction, and increased susceptibility to other illnesses.

**Figure 4:**
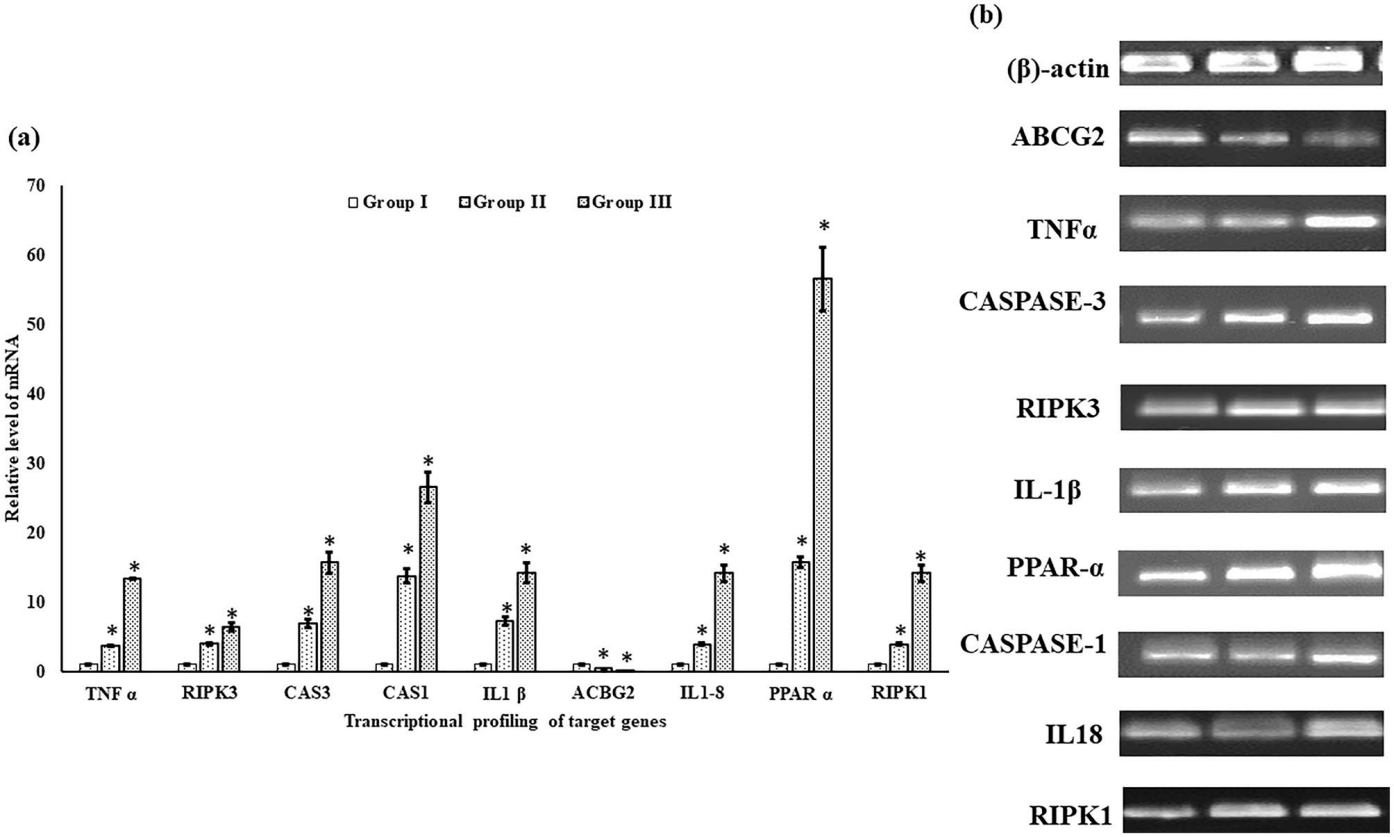
(**a**) AnRT-PCR analysis of the expression of transcripts of genes, TNF α, Caspase 3, RIPK 3, IL-1β, PPAR-α, Caspase-1, IL-18, and RIPK1 in HgCl_2_exposed liver of fish *C. punctata* of treated groups (groups II and III) in comparison to the group I (control). (**b**) The agarose gel electrophoresis image shows the band intensities of individual genes in comparison to β- actin (mean ± SD, n = 3 fishes of three replicates of each group) (asterisk represents the significant (p < 0.05) difference from the control). The original gel images are presented in Supplementary Fig S1.

After 45 days of exposure to HgCl_2_, the mRNA expression of target genes in fish of all three groups was calculated by qRT-PCR with the 2^−∆∆CT^ method (Fig. 4a). This study promulgates that expression of most of the studied genes (TNF α, Caspase 3, RIPK 3, IL-1β, PPAR-α, Caspase-1, IL-18, and RIPK1)excluding ABCG2 were increased in a time-dependent manner in HgCl_2_exposed fish in comparison to unexposed fish. In the liver of HgCl_2_treated fish, the mRNA transcripts of genes TNF α, Caspase 3, RIPK 3, IL-1β, PPAR-α, Caspase-1, IL-18, and RIPK1 were significantly (p < 0.05) induced, and the highest fold change in expressions of all the above-mentioned genes were found as 13.44, 15.7, 6.4, 14.2, 56.5, 26.5, 14.1and 14.14respectively in the liver tissue of 45 days exposed fish of group III in comparison to control. Moreover, ABCG2 mRNA level was significantly (p < 0.05) downregulated in the group III fish in comparison to the group I. However, PCR products separated by agarose gel electrophoresis exhibit changes in band intensities of genes TNF α, Caspase 3, RIPK 3, IL-1β, PPAR-α, Caspase-1, IL-18, and RIPK1 involved inHgCl_2_induced necroptosis and inflammation (Fig. 4b). For the normalization of data of genes, the β-actin gene was used as an internal control.

**Figure 5:**
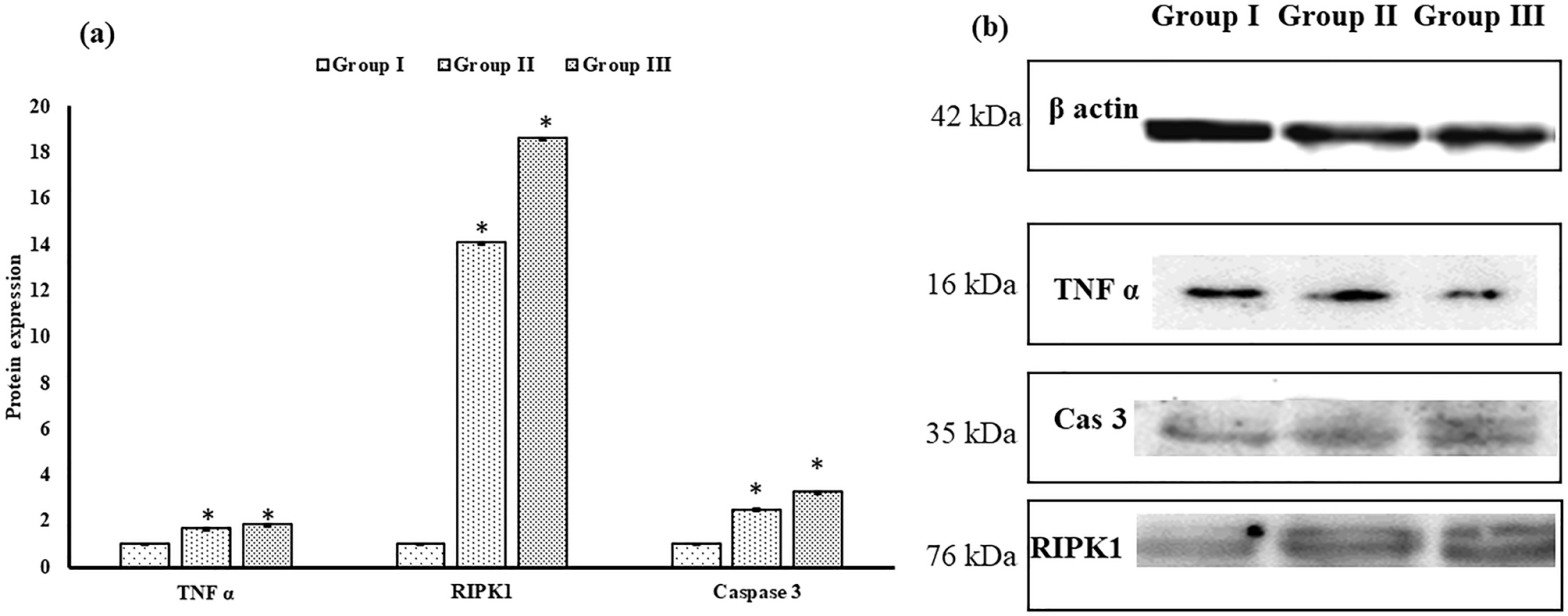
**(a)** The graph represents the quality density value based on ImageJ software with relevant β-actin as a control. (**b**) Expression of TNF α, RIPK1, and caspase 3 protein in liver tissues treated with respective treatment groups at 45 days by western blotting. Data are shown as mean ± SEM of three independent experiments (asterisk represents the significant (p < 0.05) difference from the control). The original blot images are presented in Supplementary Fig S2.

Immunoblot study showed that group III had the highest concentration of RIPK1, TNF α, and caspase-3 protein in the liver (Fig. 5a). In each of the treated groups, except the control group, HgCl_2_ caused an increase in RIPK1, caspase-3, and TNF α protein. The HgCl_2_-induced increment in aforesaid mentioned protein level was the highest in group III exposed with the highest concentration of HgCl_2_. The distribution of TNF α, RIPK1, and caspase 3 proteins are shown in Fig. 5a, b for both control and treated groups.

This study also evaluated HgCl_2_ exposure induced necroptosis and inflammation in the liver tissue, associated with oxidative stress. There is very little information present on oxidative stress induced necroptosis and inflammation in the fish liver due to exposure to mercuric chloride. This study tries to fill this gap and evaluate the oxidative potential of HgCl_2._The protein profile, and mRNA expressions of genes involved in necroptosis (TNF α, Caspase 3, RIPK 3, and RIPK1) and inflammation (IL-1β, Caspase-1, and IL-18) were measured in liver tissue of *C. punctata*, treated with different concentrations of HgCl_2_to analyze the effects of potential oxidative stress inducers on necroptosis and inflammation. Overall protein profile and mRNA expressions were increased in the liver and revealed that the increased oxidative stress may lead to damage to the cell and its protein in test fish. In an aquatic ecosystem, fish often play important roles in nutrient cycling and as prey for other organisms. Liver damage and increased inflammation in fish can have cascading effects on the ecosystem by disrupting these ecological interactions^44^.

The onset of oxidative stress, triggered by mercuric chloride exposure in fish *Channa punctata*, initiates a cascade of molecular responses. This study delves into the intricate connection between oxidative stress and the gene expression of necroptosis and inflammation-related genes. As ROS disrupt cellular homeostasis, the upregulation of genes associated with necroptosis (such as RIPK1 and RIPK3) and inflammation (including TNF α, Caspase-3, IL-1β, and IL-18) signifies molecular pathway activation. This interplay sheds light on the intricate molecular mechanisms underlying the impact of oxidative stress on the transcriptional profile, providing valuable insights into the fish's cellular responses to environmental mercury contamination. One of the largest families of membrane proteins comprises ATP-binding cassette (ABC) transporters that translocate numerous substrates and heavy metals across the plasma membrane. Amongst the ABC transporters, ABCG2 is recognized as an active transporter for heavy metals. ABCG2 gene can transport HgCl_2_ in the cells^45^ and the downregulation of ABCG2 in this study is responsible for the efflux of HgCl_2_ in the liver, Similarly, Liu et al., (2016)^46^ documented that decrement in the mRNA expression of ABCG2 transporters may control Cd transport across the placenta. It is well established that inflammation is responsible for enhancing ROS generation resulting in oxidative stress and is thought to be responsible for HgCl_2_-induced liver toxicity. In this study, the intoxication of HgCl_2_ significantly (p < 0.05) increased protein levels and mRNA transcripts of TNF-α, IL-1β, Caspase-1, and IL-18 levels, reflecting inflammatory responses. Similarly, HgCl_2_ induced hepatic damage in rats by interfering with oxidative stress and inflammation^47^. Some ligands when attached to TNF family death domain receptors can activate necroptosis. In this study, HgCl_2_ often triggers downstream key proteins, including RIPK1 and RIPK3. Specifically, RIPK1activates RIPK3 then phosphorylation of Mixed Lineage Kinase Domain-Like Pseudokinase (MLKL) takes place and is translocated to the cell membrane where it induces pore opening and necroptosis^48^. Similarly, Zhang et al., (2017)^49^ documented mitochondria derived ROS promoted oxidative stress induced automatic addition of phosphorus groups to RIPK1, this instigates RIPK1 to form necrosome with the help of RIPK3, resulting in the start of TNFα-mediated necroptosis in colon cancer cells. Numerous studies testified that heavy metals could induce cell death pathways in hepatocytes^20,28,31,35,50,51^. The cell death induced by mercuric chloride can provoke an inflammatory response, contributing to further damage and impairing liver function. Fish play integral roles in aquatic ecosystems, and liver damage can disrupt these ecosystems by affecting nutrient cycling and the food web^52^. However, it is still arcane whether HgCl_2_ can induce necroptosis in the liver of fish or not and its mechanisms involved in oxidative stress mediated necroptosis and inflammation remain to be clarified. Also, this is the first study in fish *C. punctata* that documents the role of PPARα against HgCl_2_-induced oxidative stress mediated necroptosis and inflammation. PPARαis highly expressed in vital organs such as the liver^53^. The expression of PPARα is closely related to oxidative stress and LPO. Therefore, the significant (p < 0.05) upregulation of PPARα in this study may reduce the overload of oxidative stress and also suggest an increase in oxidized fatty acids^54^. PPARα nuclear receptors regulate the expression of enzymes and proteins involved in inflammation. So, once PPARα gets activated it prevents inflammation in the liver tissue of fish. There is an ample number of data on the alleviating capacity of PPAR-α against oxidative stress induced inflammation^53,55,56^ but still a lot more has to be done for the aquatic organism.

**Figure 6:**
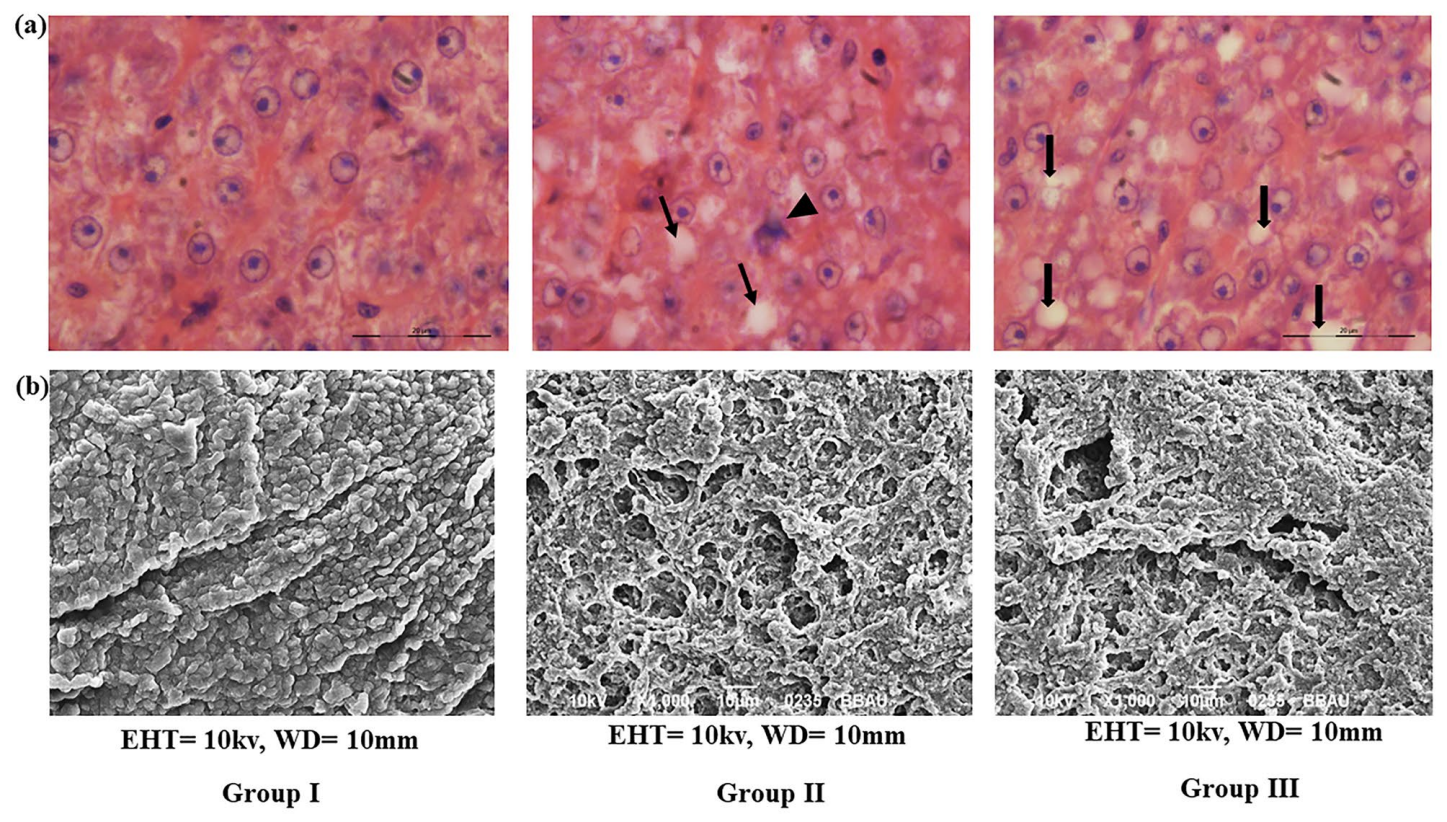
** (a)** Photomicrographs of representative hematoxylin and eosin-stained liver sections prepared from control fish and fish treated with two sublethal concentrations of HgCl_2_ for 45 days. A ×100 magnification of group II and group III liver sections reveal a focus of inflammatory cells by an arrowhead. Hepatocytes containing micro steatosis are denoted with small arrows, whereas big arrows designate macro steatosis. (**b**) Scanning electron microscope images showing micro steatosis in the liver of group II and mega steatosis in group III intoxicated with HgCl_2_.

Scanning electron microscope images and stained microphotographs of control as well as treated fish tissue are depicted in Fig. 6a, b. Liver tissue of control fish displayed normal histology and morphology, whereas micro steatosis and macro steatosis were prominent in group II and group III, treated with HgCl_2_. The uneven fat deposition was recorded among the allHgCl_2_ treated groups; defined foci of micro steatosis were most prominent in areas of the hepatic lobule of group II fish liver tissue. However, macro steatosis appeared to be a more common feature among the fish treated with the highest concentration of HgCl_2_. The100× images display representative foci of inflammatory cells scattered among regions of micro- and macro-steatosis present in HgCl_2_ treated groups. The SEM facilitated the observation of micro steatosis and macro steatosis in the HgCl_2_ treated liver (Group II and III).

The augmented expression of necroptosis and inflammation-related genes in response to mercuric chloride exposure, as observed in this study, establishes a noteworthy link to the development of liver steatosis in fish. The upregulation of genes such as RIPK1, RIPK3, TNF α, Caspase-3, IL-1β, and IL-18 signifies a molecular orchestration that contributes to the observed pathological changes. This study unveils a direct correlation between heightened gene expression and the manifestation of liver steatosis, providing a comprehensive understanding of the molecular mechanisms underpinning the adverse effects of mercury contamination on fish liver health. Liver injury and its severity are directly proportional to regulated cell death. Hepatocyte death is a critical event in the progression of liver disease, ultimately leading to fibrosis due to the resultant inflammation. The pathogenesis of various liver disorders is a consequence of apoptosis, necrosis, necroptosis, steatosis, autophagy, pyroptosis, and ferroptosis. In this study, HgCl_2_ increases the accumulation of lipids leading to liver steatosis. Similarly, an increase in severe liver lesions/damage was reported due to enhanced hepatic steatosis in animals exposed to Cd^57^. Moreover, it is well established that Hg exposure increases the susceptibility of the liver to oxidative stress mediated steatosis in zebrafish^58^. Fish with liver steatosis may suffer from poor growth, weakened immune responses, and increased susceptibility to other diseases and stressors. Moreover, Liver steatosis can affect reproductive capabilities, potentially leading to decreased fish population size^59^. Liver steatosis induced by heavy metals in fish can disrupt nutrient cycling within aquatic ecosystems, affecting the availability of essential nutrients for other organisms^53,60^. Changes in fish populations and their health can have cascading effects throughout the food web, potentially leading to imbalances and reduced biodiversity.

Evaluation by correlation among targeted parameters exhibited a strong connotation of physiological and molecular perturbations with higher correlation coefficient (R) values in the liver tissue of HgCl_2_ exposed fish. Precisely, the increased generation of ROS causes oxidative stress which is evident by an enhancement in lipid peroxidation and protein carbonyl activity in hepatic tissue. Therefore, a strong positive correlation was detected amid ROS and LPO, protein carbonyl, SGOT, and SGPT in the liver of fish treated with HgCl_2_ (Supplementary Table 1). Furthermore, a strong positive correlation between ROS and LPO, TNF α, Caspase 3, RIPK 3, IL-1β, PPAR-α, Caspase-1, IL-18, and RIPK1 was found in the liver treated with HgCl_2_. The relationship between ROS, LPO, and protein levels of TNF α, caspase-3, and RIPK1 was analyzed to examine the relationship between oxidative stress, necroptosis, and inflammation in the test fish. The correlation between transcripts of mRNA and the protein level of targeted genes of necroptosis and inflammation showed a strong positive correlation in the HgCl_2_ intoxicated fish, *Channa punctata* (Supplementary Table 2).

This study provides crucial insights into the physiological and molecular responses associated with oxidative stress, necroptosis, and inflammation in the liver of a freshwater food fish. The findings reveal significant alterations in ROS production, lipid peroxidation, liver marker enzymes, and the expression of genes related to necroptosis and inflammation, shedding light on the intricate mechanisms of mercury-induced toxicity. The study's application lies in addressing the urgent environmental and public health concerns related to mercury contamination in aquatic ecosystems, particularly in India. By elucidating the pathways involved in liver steatosis and the molecular responses triggered by HgCl_2_, this research contributes valuable information for formulating effective policies to mitigate mercury pollution, safeguarding fish populations, and ultimately preserving the ecological balance of aquatic ecosystems. The documented correlations between oxidative stress, necroptosis, and inflammation underscore the interconnectedness of these processes, emphasizing the need for comprehensive approaches in addressing the multifaceted challenges posed by mercury contamination.

## Conclusion

**Figure 7:**
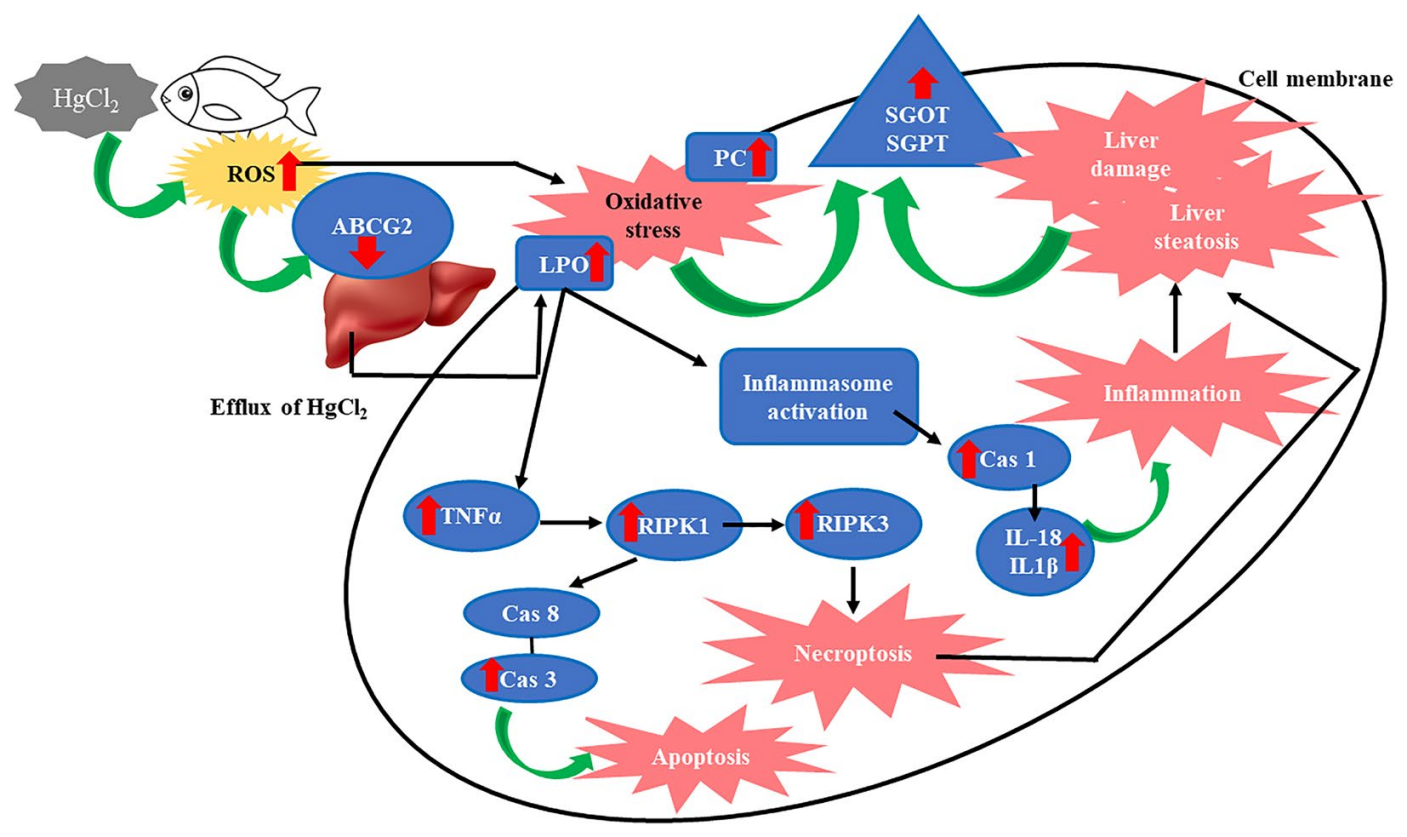
Schematic diagram to show the probable pathways of mercuric chloride-induced oxidative stress mediated necroptosis, inflammation, and liver steatosis in fish, *Channa punctata.*

The findings of the study were analyzed and interpreted in the context of the research gaps. The authors highlighted the significant increase in oxidative stress markers, the presence of necroptosis and inflammation, and the occurrence of liver steatosis as a result of HgCl_2_ exposure. The upregulation of PPARα was interpreted as the fish's response to counteract the effects of liver steatosis. The findings of the study reveal that HgCl_2_ exposure induced oxidative stress, lead tonecroptosis and inflammation in the liver of fish *C. punctata* through the regulation of TNF α/ RIPK1/RIPK3/Caspase 3/ Caspase 1/ IL1-β/IL18 signaling (Fig. 7). This study establishes a link between various consequences of HgCl_2_ exposure in the fish liver, emphasizing the role of oxidative stress in hepatotoxicity. Further research is needed to fully understand the mechanism of PPARα in mitigating heavy metal toxicity. The study holds practical applications in environmental protection, public health, fisheries management, and policy development, addressing the critical issue of mercury contamination in aquatic ecosystems and its implications for human health.

### Supplementary Information

Supplementary Information.

## Data Availability

All data generated or analyzed during this study are included in this manuscript (and its Supplementary Information files).

## Abbreviations

HgCl_2_: Mercuric chloride
ROS: Reactive oxygen species
LPO: Lipid peroxidation
PC: Protein carbonyls
SGOT: Serum glutamic-oxaloacetic transaminase
SGPT: Serum glutamic pyruvic transaminase
ABCG2: ATP-binding cassette subfamily G member 2
TNF α: Tumour necrosis factor-alpha
RIPK 3: Receptor-interacting protein kinase 3
Cas-3: Caspase-3
IL-1β: Interleukin-1β
IL-18: Interleukin-18
Cas-1: Caspase-1
RIPK1: Receptor-interacting protein kinase 1
PPAR α: Peroxisome proliferator-activated receptor alpha
LC: Lethal concentration
KMNO_4_: Potassium per magnet
APHA: American Public Health Association
OECD: Organization for Economic Co-operation and Development
EDTA: Ethylenediaminetetraacetic acid
NADH: Nicotinamide adenine dinucleotide (NAD) + hydrogen (H)
NAD: Nicotinamide adenine dinucleotide
RNA: Ribonucleic acid
cDNA: Complementary deoxyribonucleic acid
