# Supplementary material for: Oxidative stress, inflammation, and steatosis elucidate the complex dynamics of HgCl2 induced liver damage in Channa punctata
Source: Sci Rep. 2024 Apr 22;14:9161. doi: 10.1038/s41598-024-59917-4 (PMC11033285; doi:10.1038/s41598-024-59917-4)

TITLE PAGE

Oxidative stress, inflammation, and steatosis elucidate the complex dynamics of $HgCl_2$ induced liver damage in *Channa punctata*

Shefalee Singh[1], Shikha Dwivedi[1], Adeel Ahmad Khan[1], Anamika Jain[1], Shraddha Dwivedi[4] Kamlesh Kumar Yadav[3], Indrani Dubey[2], Abha Trivedi[5], Sunil P. Trivedi[1], Manoj Kumar[1*]

[1]Environmental Toxicology & Bioremediation Laboratory (ETBL), Department of Zoology, University of Lucknow, Lucknow- 226007 (India)
[2]Department of Zoology, DBS College, Kanpur, Uttar Pradesh-208006 (India)
[3]Department of Zoology, Government Degree College, Bakkha Kheda, Unnao-209801 (India)
[4]Department of Zoology, Government Degree College, Haripur-Nihastha, Raebareli - 229208 (India)
[5]Department of Zoology, Mahatma Jyotiba Phule Rohilkhand University, Bareilly, Uttar Pradesh-243006 (India)
*Corresponding author: Dr. Manoj Kumar, mk2016lu@gmail.com

Ms. Shefalee Singh: Research Scholar, Environmental Toxicology & Bioremediation Laboratory, Department of Zoology, University of Lucknow, Lucknow- 226007 (India) Email id: shefalee.singh@gmail.com

Ms. Shikha Dwivedi: Research Scholar, Environmental Toxicology & Bioremediation Laboratory, Department of Zoology, University of Lucknow, Lucknow- 226007 (India)Email id: shikha.dwivedi0000@gmail.com

Mr. Adeel Ahmad Khan: Research Scholar, Environmental Toxicology & Bioremediation Laboratory, Department of Zoology, University of Lucknow, Lucknow-226007 (India). Email id: adeelkhan756@gmail.com

Ms. Anamika Jain: Research Scholar, Environmental Toxicology & Bioremediation Laboratory, Department of Zoology, University of Lucknow, Lucknow-226007 (India). Email id: anamika.zoo0811@gmail.com

Dr. Shraddha Dwivedi: Department of Zoology, Government Degree College, Haripur-Nihastha, Raebareli - 229208 (India). Email id: shraddhadwivedi2013@gmail.com

Dr. Kamlesh Kumar Yadav: Department of Zoology, Government Degree College, Bakkha Kheda, Unnao-209801 (India). Email id: drkkyadav8@gmail.com

Dr. Indrani Dubey: Department of Zoology, DBS College, Kanpur, Uttar Pradesh-208006 (India) Email id: indranidubey31@gmail.com

Dr. Abha Trivedi: Department of Zoology, Mahatma Jyotiba Phule Rohilkhand University, Bareilly, Uttar Pradesh- 243006 (India). Email id: abha14sep@gmail.com

Dr. Sunil P. Trivedi: Professor, Department of Zoology, University of Lucknow, Lucknow-226007(India) sat060523@gmail.com.

*Dr. Manoj Kumar: Assistant Professor, Department of Zoology, University of Lucknow, Lucknow-226007 (India) Email id: mk2016lu@gmail.com**Corresponding Author.**

Supplemetary Figure S1: Original image for figure 4 (b)

Figure 4 b

Original image for IL18 and RIPK1

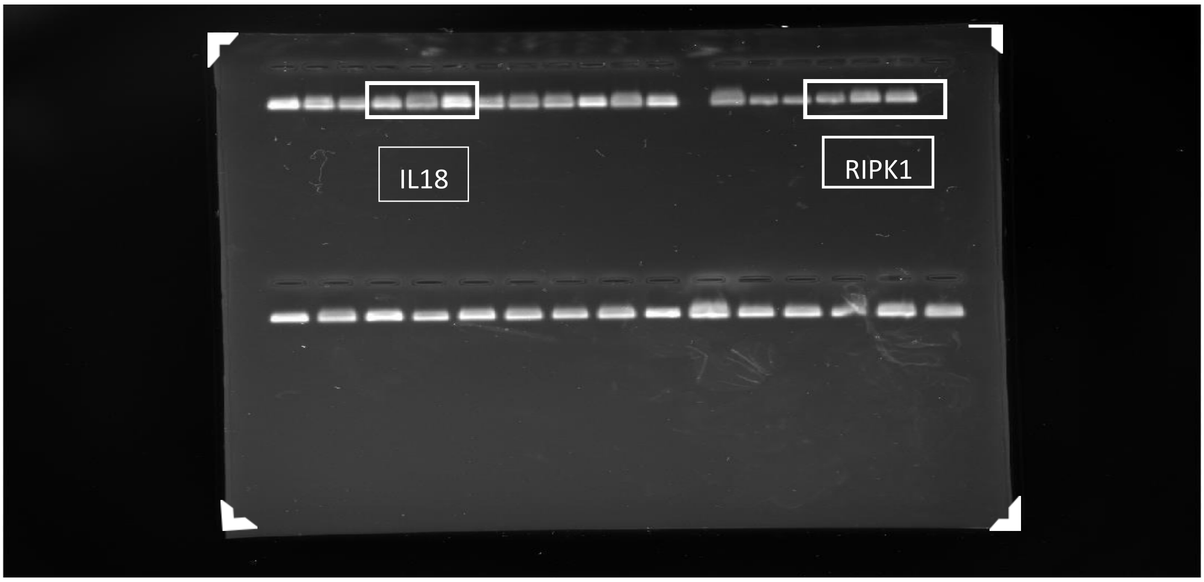

Figure 4 b

Original image for ABCG2

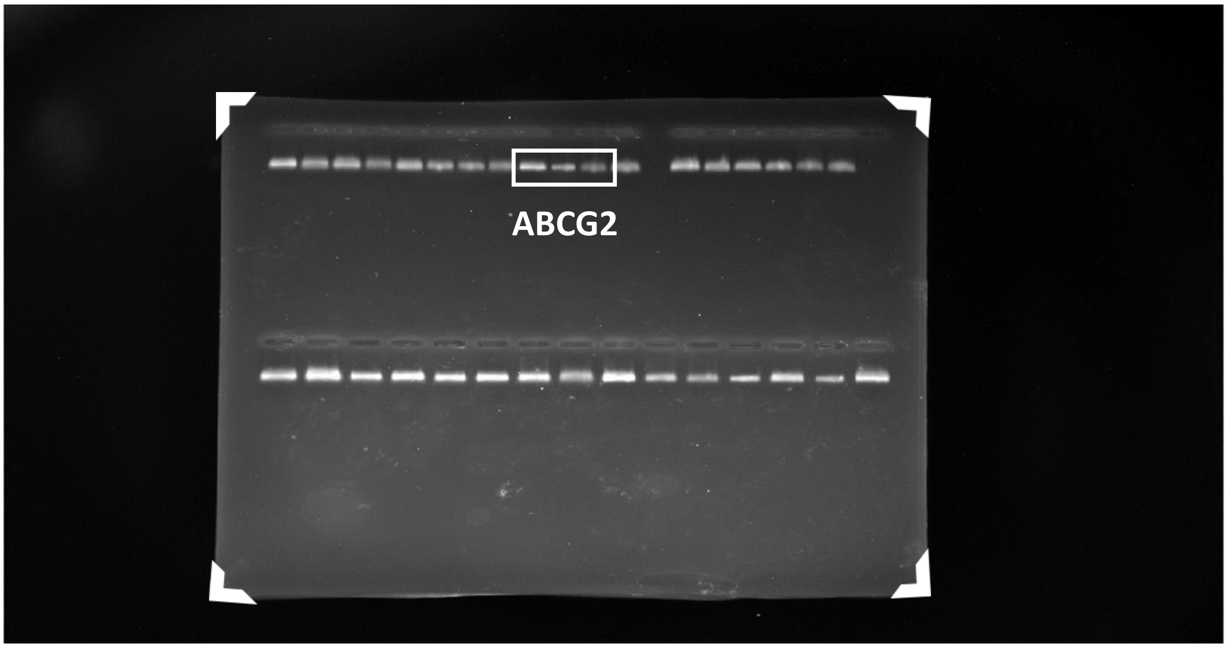

Figure 4 b

Original image for TNF α

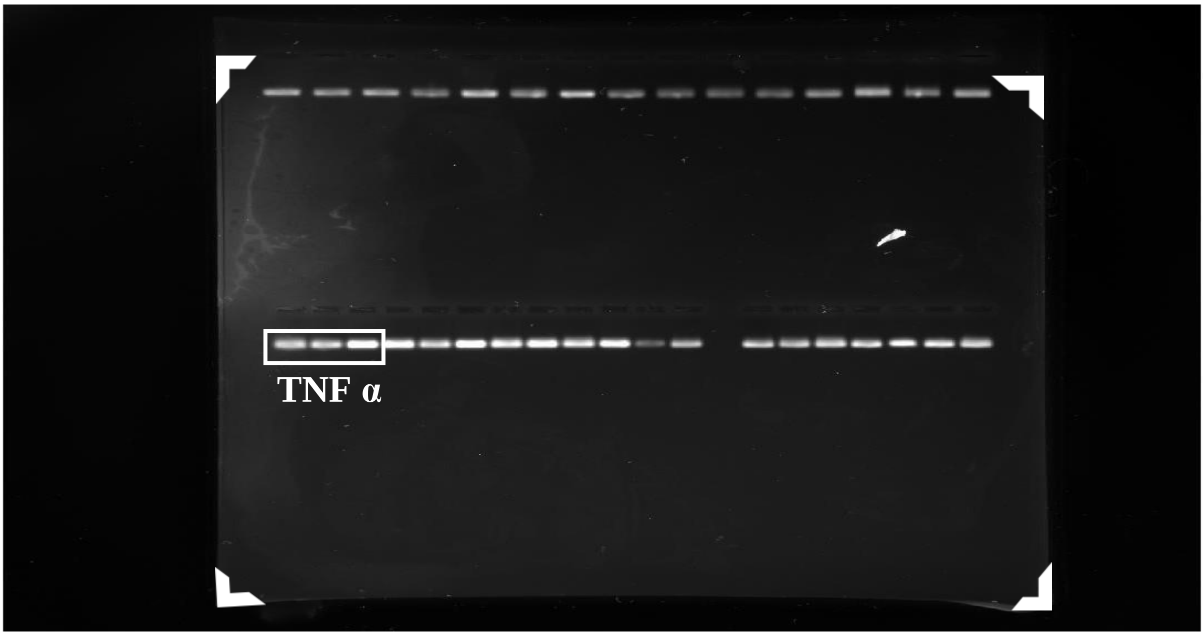

Figure 4 b

Original image for Caspase 3

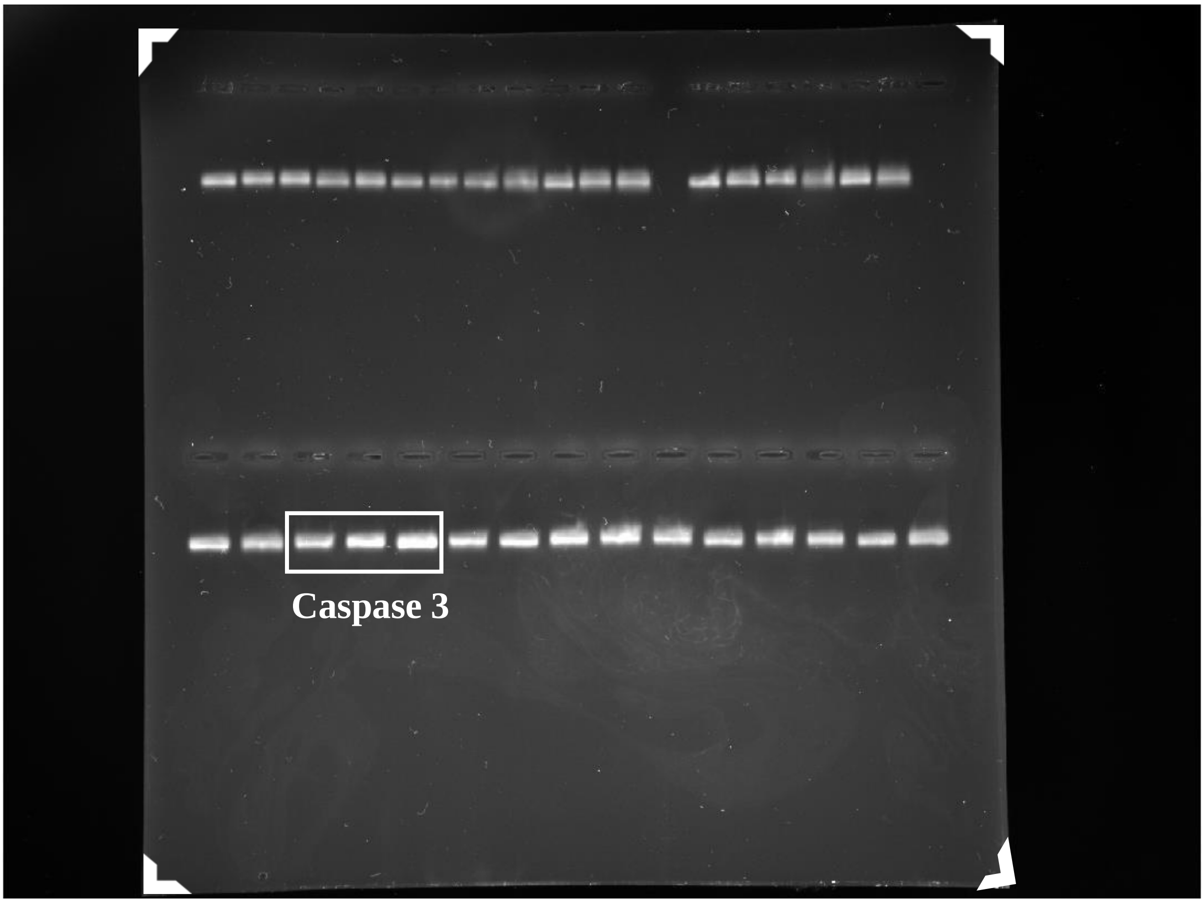

Figure 4 b

Original image for PPAR α

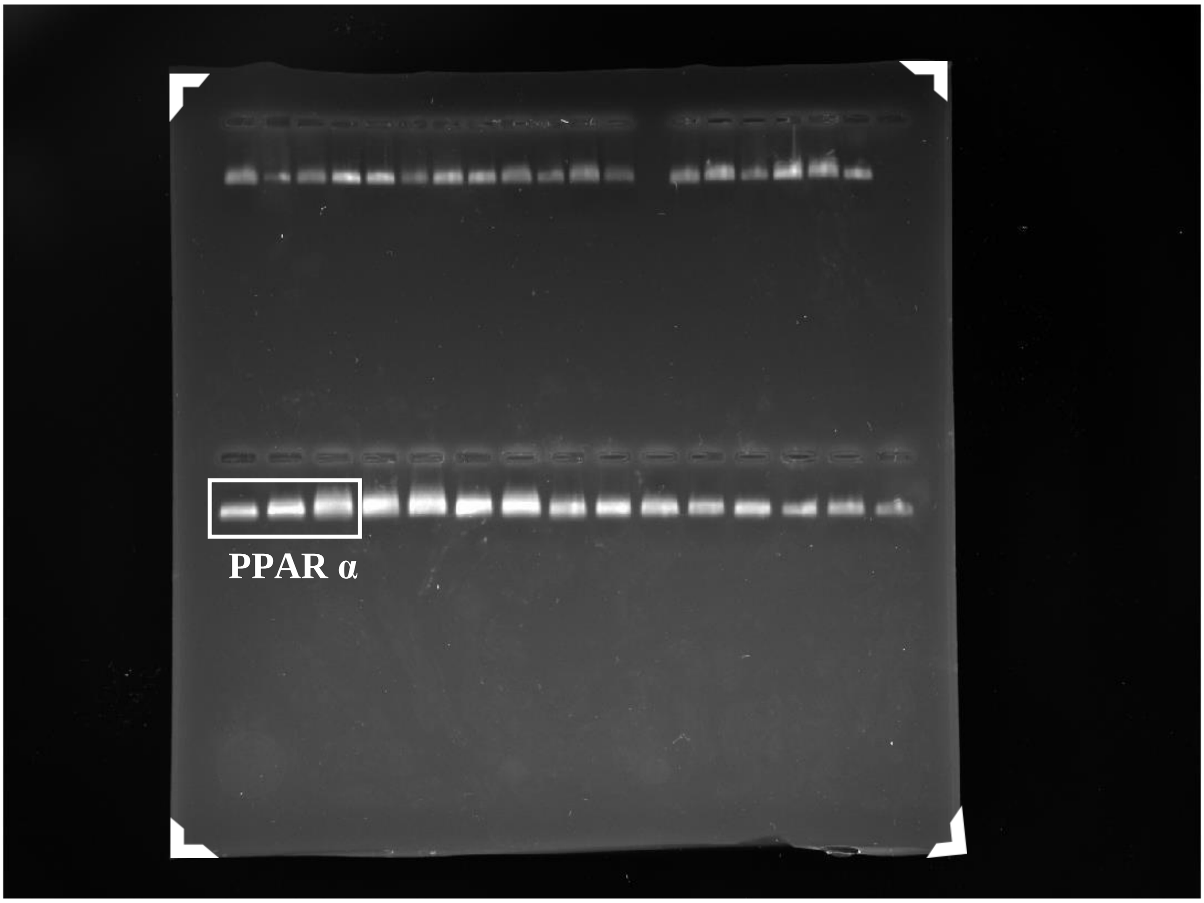

Figure 4 b

Original image for Caspase 1

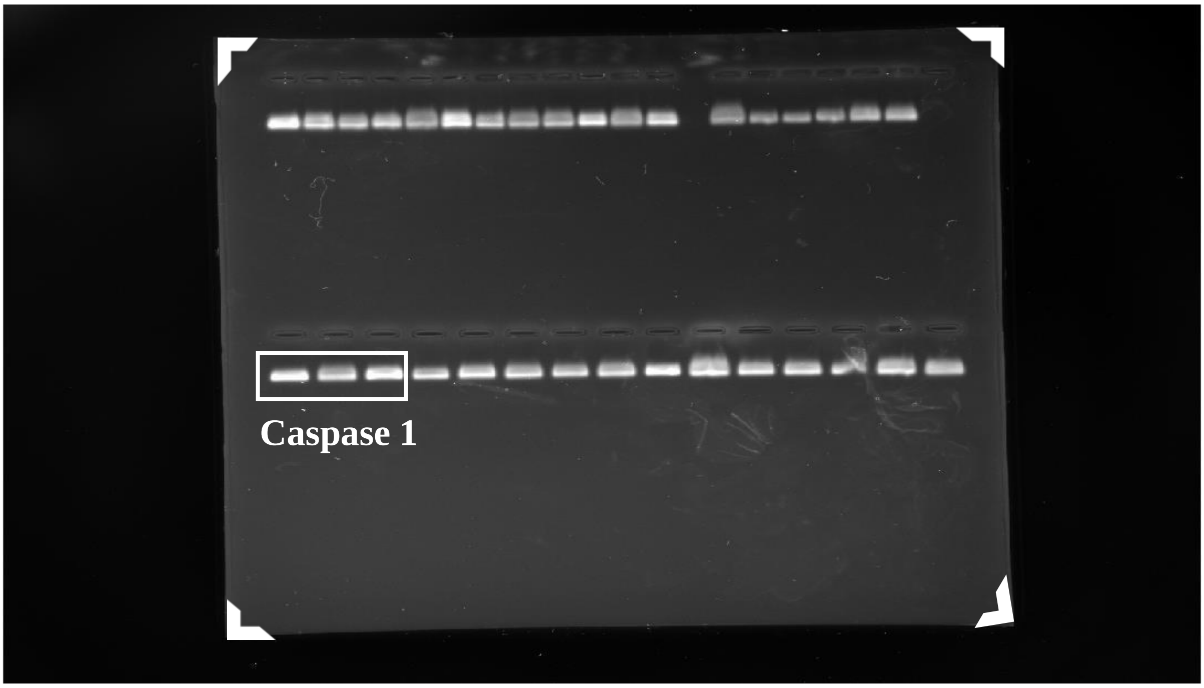

Figure 4 b

Original image for β Actin

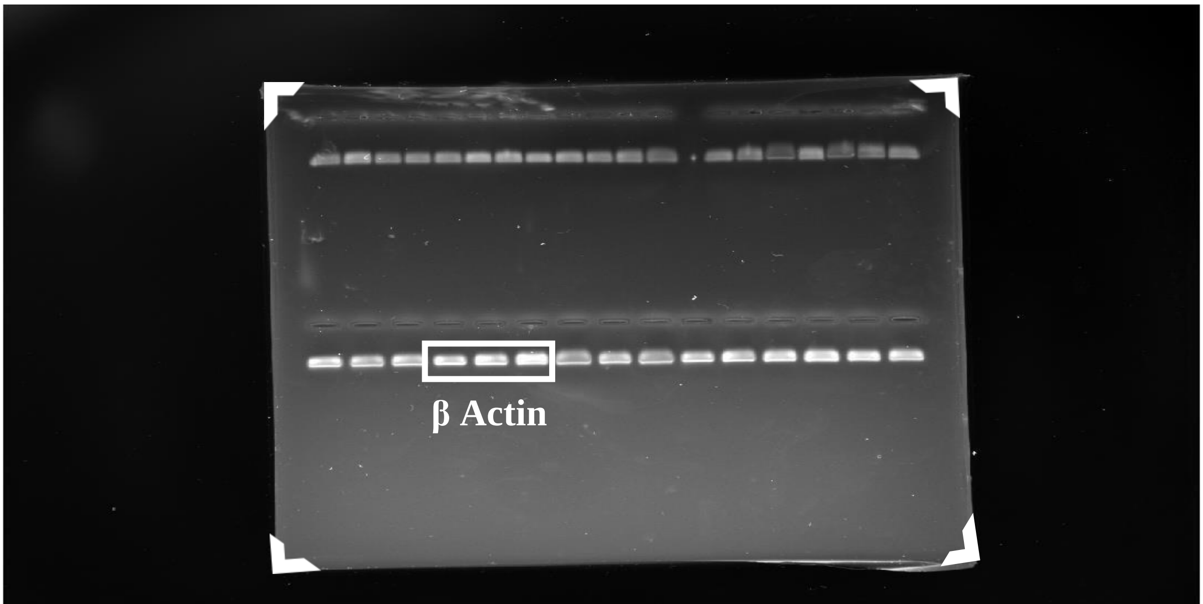

Supplemetary Figure S2: Original image for figure 5 (b)

Figure 5 b

Original image for β Actin

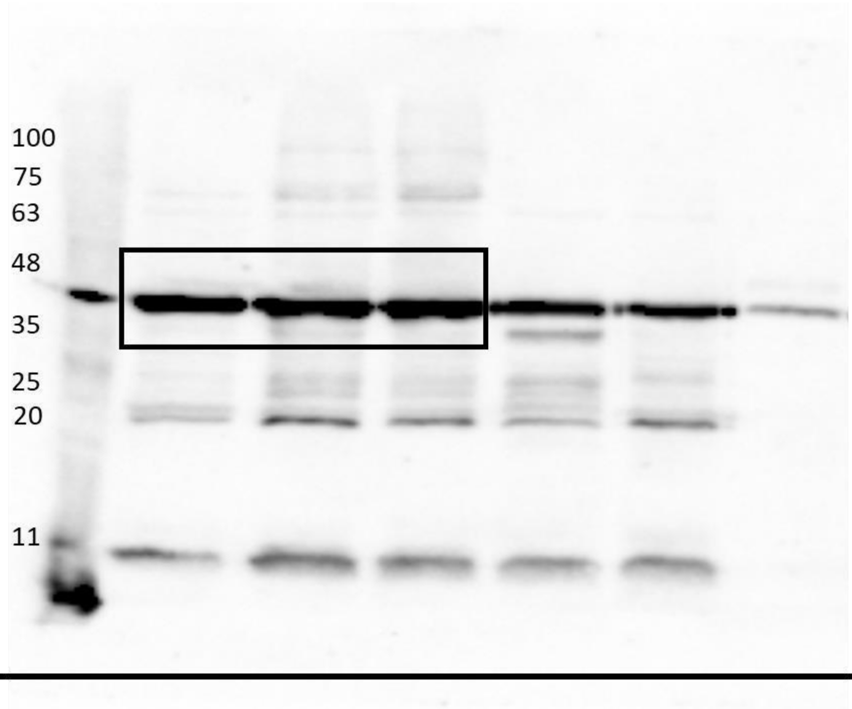

Figure 5 b

Original image for TNF α

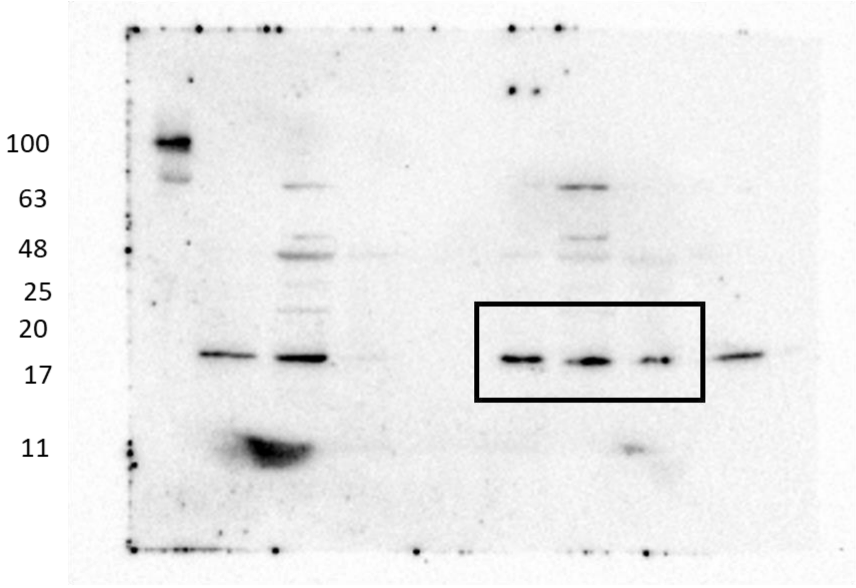

Figure 5 b

Original image for RIPK1

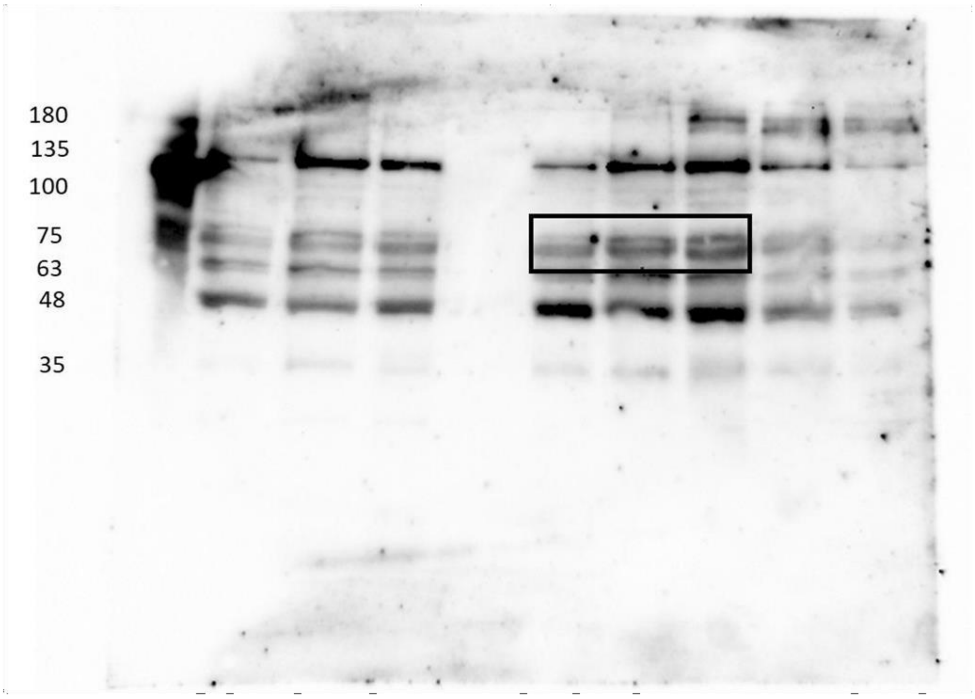

Figure 5 b

Original image for Caspase 3

The full length immune blot for caspase 3 could not be provided because blots were cut prior to hybridization with antibodies

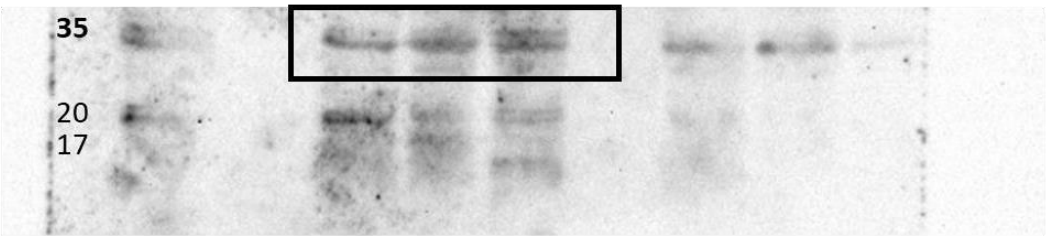

Table 1 Supplementary material.

Correlation coefficients by linear regression analysis between oxidative stress markers and liver marker enzymes

	ROS	*LPO*	*PC*	*SGOT*	*SGPT*
ROS	1**				
LPO	0.221377**	1**			
PC	0.918668**	0.181857**	1**		
SGOT	0.110024**	0.944911**	0.493707**	1**	
SGPT	0.805756**	0.755929**	0.506266**	0.5**	1**

Table 2 Supplementary material.

Correlation coefficients by linear regression analysis between oxidative stress markers and target genes of necroptosis and inflammation.

	ROS	LPO	TNF	RIPK3	IL1B	IL18	CAS 1	CAS3	RIPK1	ACBG2	PPAR	CAS 3 Protein	RIPK1 Protein	TNF α Protein
ROS	1**													
LPO	0.22138	1**												
TNF	1**	-0.22138	1**											
RIPK3	-0.22138	1**	-0.22138	1**										
IL1B	1**	-0.22138	1**	-0.22138	1**									
IL18	-0.22138	1**	-0.22138	1**	-0.22138	1**								
CAS 1	0.692099**	0.550679**	0.692099**	0.550679**	0.692099**	0.550679**	1**							
CAS3	0.745328**	0.485158**	0.745328**	0.485158**	0.745328**	0.485158**	0.997065**	1**						
RIPK1	0.760353**	0.46507**	0.760353**	0.46507**	0.760353**	0.46507**	0.995057**	0.999739**	1**					
ACBG2	0.702647**	0.538335**	0.702647**	0.538335**	0.702647**	0.538335**	0.999892**	0.998084**	0.996411**	1**				
PPAR	0.760353**	0.46507**	0.760353**	0.46507**	0.760353**	0.46507**	0.995057**	0.999739**	1**	0.996411**	1**			
CAS 3 Protein	-0.14757	0.99718**	-0.14757	0.99718**	-0.14757	0.99718**	0.611764**	0.549408**	0.53019**	0.600056**	0.53019**	1**		
RIPK1 Protein	-0.19368	0.999599**	-0.19368	0.999599**	-0.19368	0.999599**	0.574089**	0.509718**	0.489945**	0.561976**	0.489945**	0.99890515**	1**	
TNF α Protein	0.260531**	0.883835**	0.260531**	0.883835**	0.260531**	0.883835**	0.877189**	0.837855**	0.825175**	0.870028**	0.825175**	0.916447124**	0.896723895**	1**

Supplementary Figure S3: Trypan blue staining. Microscopic images at 40X magnification of the blood cells in Group I and mercuric chloride treated groups (Group II and Group III) after 15, 30 and 45 days. The cells were stained using trypan blue dye after mercuric chloride exposure to examine cell damage. The cells indicated by arrows are some examples of dead cells having damaged cell membrane and turned dark blue after staining. (Scale bar 100µm).

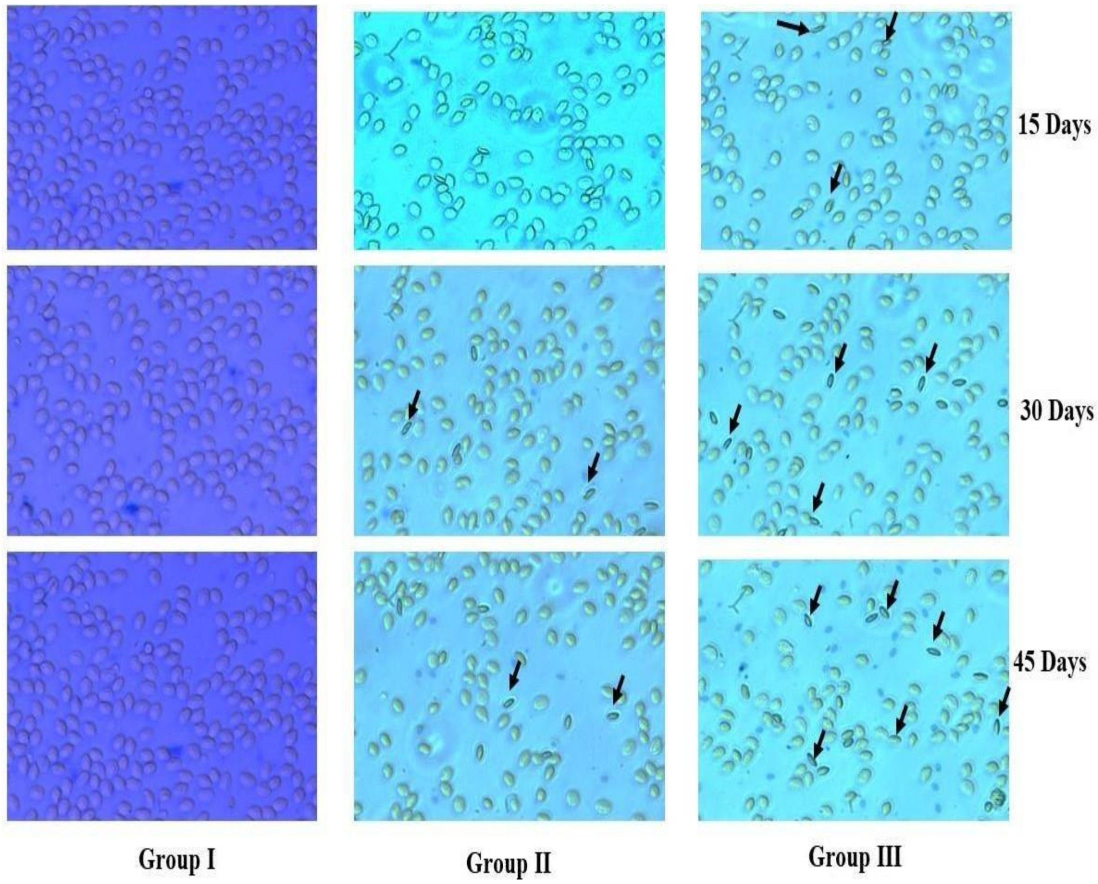

Supplement: Supplementary file 1 — Supplementary Information. [file 41598_2024_59917_MOESM1_ESM.pdf]
